# Human *Ex-Vivo* Liver Model for Acetaminophen-induced Liver Damage

**DOI:** 10.1038/srep31916

**Published:** 2016-08-23

**Authors:** Thomas Schreiter, Jan-Peter Sowa, Martin Schlattjan, Jürgen Treckmann, Andreas Paul, Karl-Heinz Strucksberg, Hideo A. Baba, Margarete Odenthal, Robert K. Gieseler, Guido Gerken, Gavin E. Arteel, Ali Canbay

**Affiliations:** 1 Department for Gastroenterology and Hepatology, Center for Internal Medicine, University Hospital, University Duisburg-Essen, Essen, Germany; 2 Department of General, Visceral and Transplantation Surgery, University Hospital, University Duisburg-Essen, Essen, Germany; 3 Department of Clinical Chemistry, University Hospital, University Duisburg-Essen, Essen, Germany; 4 Institute of Pathology, University Hospital, University Duisburg-Essen, Essen, Germany; 5 Institute of Pathology, University Hospital Cologne, Cologne, Germany; 6 Rodos BioTarget GmbH, Medical Park Hannover, Hannover, Germany; 7 University of Louisville Health Sciences Center, Louisville, Kentucky, USA

## Abstract

Reliable test systems to identify hepatotoxicity are essential to predict unexpected drug-related liver injury. Here we present a human *ex-vivo* liver model to investigate acetaminophen-induced liver injury. Human liver tissue was perfused over a 30 hour period with hourly sampling from the perfusate for measurement of general metabolism and clinical parameters. Liver function was assessed by clearance of indocyanine green (ICG) at 4, 20 and 28 hours. Six pieces of untreated human liver specimen maintained stable liver function over the entire perfusion period. Three liver sections incubated with low-dose acetaminophen revealed strong damage, with ICG half-lives significantly higher than in non-treated livers. In addition, the release of microRNA-122 was significantly higher in acetaminophen-treated than in non-treated livers. Thus, this model allows for investigation of hepatotoxicity in human liver tissue upon applying drug concentrations relevant in patients.

Potential hepatotoxicity is a key issue in newly developed, repurposed and approved drugs. Drug-induced liver injury (DILI) has mostly been investigated in animal models or specialized cell culture systems. These often fail to reflect all relevant metabolic processes in the human *in-vivo* setting^1^. In contrast, reliable *ex-vivo* systems of functionally intact human liver tissue promise to avoid such shortcomings and may even outperform the informative value of animal models. Such systems might be able to complement animal toxicity studies prior submitting a clinical trial application.

We earlier introduced an *ex-vivo* system that allows emulation of *in-vivo* conditions in noncirrhotic and cirrhotic human liver tissue by perfusing human liver sections for six hours^2^. The perfusion time could be extended to 30 h by adaptations in composition of the perfusion medium and assembly of the system (see Fig. 1a).

In industrialized countries, acetaminophen (APAP) intoxication is a major reason for acute liver failure (ALF). Within the hepatocyte a small amount of APAP is metabolized to N-Acetyl-p-benzochinonimin (NAPQI) by cytochrome P450 isoenzyme 2E1 (CYP2E1). NAPQI modifies functional proteins in the mitochondria leading to mitochondrial damage and subsequent cell necrosis^3^,^4^. The well-characterized damage pattern of acetaminophen allows its use to evaluate performance of DILI models^5^. In this study perfused liver sections were treated with acetaminophen to establish a possible use of the perfusion system as model for DILI and to identify hepatotoxicity.

## Materials and Methods

### Patients and Liver Samples

All patients (7 male, 2 female; listed in Table 1) provided written informed consent according to the Helsinki declaration of 1995. The study protocol conformed to the guidelines of and was approved by the ethics committee of the University Hospital Essen (12-5232-BO). All procedures were carried out in accordance with the Helsinki declaration of 1995. Normal liver tissue was obtained from partial liver resections performed at the Department for General, Visceral and Transplantation Surgery. Excised liver tissue was forwarded to the pathologist as a whole; the diseased part was identified for examination and removed. Thus a section of normal tissue was retrieved for perfusion, separated with an ample circumference of surrounding intact tissue. The piece was transferred to our laboratory in cold Hanks’ balanced salt solution (HBSS) (see below: Experimental Perfusion) and stored at 4 °C until further processing.

### Liver Perfusion System

**Figure 1:**
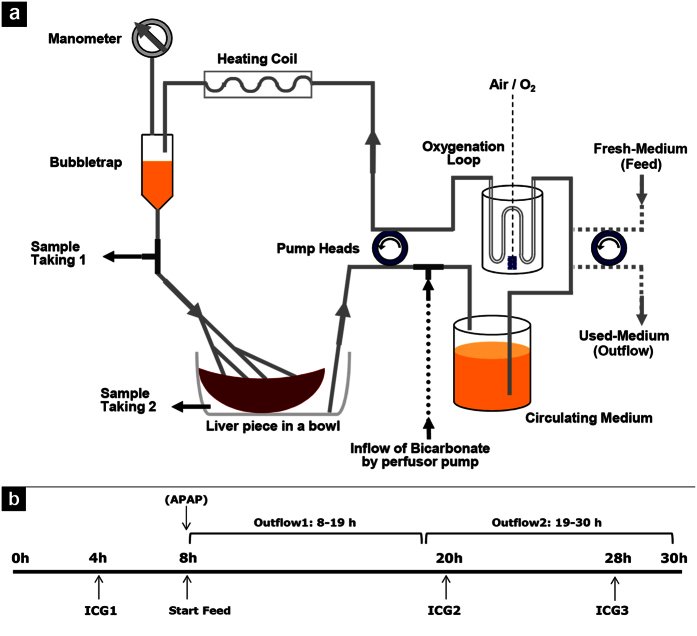
Outline of the perfusion system and time schedule for perfusion experiments. (**a**) The system enabled a circular flow of the culture medium for continuous heating, oxygenation and tissue perfusion. Simultaneous sample taking before and after the liver passage allowed for surveillance of oxygen consumption. By pH measurement and inflow of sodium bicarbonate pH was maintained in a physiological range. (**b**) Schedule of the perfusion runs with time points of liver function tests, start of extra-circular addition of fresh medium and application of APAP for toxicity experiments.

The perfusion system was designed as a closed circuit unit providing a constant flow rate for supplying liver samples of 25 to 60 g with nutrients and oxygen (for schematic see Fig. 1a). A pump drive with two peristaltic heads (Masterflex; Novodirect, Kehl, Germany) was used for the bidirectional transport of the perfusion medium. Medium (see: Experimental Perfusion) was oxygenated in a glass bottle via the thin wall of special silicon tubing and routed through a custom-built glass heating coil flowed with water kept at 40 °C via an external heating outlet (Julabo; Seelbach, Germany). The fluid’s hydraulic pressure was measured manometrically (VBM Medizintechnik; Sulz, Germany). Pressure ranged from 10–40 mm Hg at the start of perfusion experiments and reached 60 to 200 mm Hg towards the end. Before entry into the liver sample, the perfusion fluid was degassed by means of a bubble trap (Stem Cell Systems; Berlin, Germany). A three-way valve was installed thereafter to collect samples of perfusion medium before passing the liver sample. The specimen was connected via four branches of the circuit tubing, ending in venous catheters, and the perfusate efflux was collected in a bowl and reconveyed to the medium reservoir. This connection was fitted with another three-way valve to apply test agents in the course of perfusion. Liver specimens were maintained at 37 °C in a water bath.

### Experimental Perfusion

Onset of the perfusion procedure ranged from 1 to 3 h after tissue retrieval. First, the liver specimen was connected to the perfusion circuit by the tissue adhesive Histoacryl (B. Braun; Melsungen, Germany) via four cannulas into suitable vessels to minimize generation of pressure. Second, the surgical cutting area was sealed with tissue adhesive. The liver piece was then rinsed with approximately 500 mL of HBSS (Biochrom; Berlin, Germany) supplemented with 20 mM 4-(2-hydroxyethyl)-1-piperazineethanesulfonic acid (HEPES) (GE Healthcare; Freiburg, Germany) and 2 U/mL heparin (Ratiopharm; Ulm, Germany) to remove residual blood. Subsequently, the perfusion medium was exchanged for 250 mL of Williams’ medium E (Biochrom) supplemented with 20 mM HEPES, 240 mg/dL glucose (B. Braun), 0.8% Dextran 60 EP (Pharmacosmos; Holbaek, Denmark), 0.25% β-cyclodextrin (Sigma-Aldrich; Taufkirchen, Germany), 20 mg/mL gentamicin (Biochrom), 2 mL 100x non-essential amino acids (Biochrom), 2 mL of 50x essential amino acids (Biochrom), 5 mL 100x glutamine-penicillin-streptomycin (Gibco Life Technologies; Darmstadt, Germany), and 0.08% pluronic F68 (Gibco), pH-adjusted to 7.4 with 10 N NaOH (Referred to as medium A). The first 80-mL volume of the perfusate was discarded to remove residual HBSS. The circuit was then closed by placing the effluent tubing into the medium reservoir (Fig. 1a) and perfusion was run for 2 min before initial sampling. At this point, time measurement was started, and flow rates were adjusted to obtain an appropriate pressure of approximately 20 mmHg at 35 mL/min. The perfusion was run initially for 8 h and perfusate samples were collected at hourly intervals. Glucose and lactate concentrations as well as pH and pO_2_ (general metabolism) were measured instantly on a blood gas analyzer (ABL800; Radiometer; Willich, Germany). During this starting phase, the pH was adjusted to around 7.4 by adding 1 to 2 ml of 8.4% sodium bicarbonate solution (Braun) to the reservoir as needed. At pH > 7.5, 0.1 to 0.2 mL of 1 M citric acid (Sigma-Aldrich) was added for readjustment. If continuous inflow of sodium bicarbonate became necessary, a 50 mL syringe in a perfusion device was connected to the effluent tubing with a flow setting of 0.2–3.2 mL/h (as appropriate to maintain pH). After the initial 8 h of perfusion, influx of medium B was started at about 11 mL/h via the second pump drive. Medium B contained the above described medium A plus 160 mg/dL glucose and 2 mM of, each, sodium pyruvate, 2-oxoglutarate and oxaloacetate (Sigma-Aldrich) as anaplerotic substrates. The outflow rate from perfusate was reduced to about 6 mL/h by using a tubing with a smaller inner diameter to compensate for any loss in the circuit by evaporation; the outflow 1 was collected in a glass bottle on ice (8–19 h).

For experiments with acetaminophen treatment, the adequate volume was added to the reservoir at 8 h from a stock solution of 12 mg/mL (Sigma-Aldrich) in medium A. An equal concentration was added to the inflow bottle of medium B. On the next day, hourly sampling was continued from 19 h to 30 h and the effluent bottle was replaced for the second outflow duration (19–30 h). When the glucose concentration in the perfusion solution decreased below 200 mg/dL, the perfusate was supplemented with 4 mL of a 40% glucose solution (Braun) corresponding to about 640 mg/dL glucose. Upon termination of the experiment, 2 mL of trypan blue (Sigma-Aldrich) was added and perfused for 10 min to allow identification of perfused tissue areas. Liver specimens were cut into 1-cm slices, and areas of interest were transferred to storage solutions appropriate for subsequent analyses. Figure 1b provides a simplified view of the perfusion schedule.

### Assessment of indocyanine green clearance

Liver function tests were performed at 4, 20 and 28 h of perfusion. Indocyanine green (ICG) was added to the reservoir at 0.09 mg per g of liver from a stock solution of 5 mg/mL (Pulsion Medical Systems; Feldkirchen, Germany). For testing, 550 μL of perfusate were removed. A sample taken before onset served as blank value. As described above the perfusion was initially run for 2 min, which was set as the zero point (t = 0 min). Further samples were taken at 4, 8, 12, 16, 20, 30, 50 and 90 min of perfusion. Optical densities were measured at 805 nm on a Fluostar Omega (BMG Labtech; Ortenberg, Germany) in duplicates with 250 μL samples, each. Data analysis was performed via MS Excel, where ICG half-lives were calculated as ln(2)/k from the trend curve A(t) = A_0_*e^−kt^ for the exponential phase of the curve.

### Biochemical Parameters and Calculations

To determine biochemical parameters, perfusate samples were stored at 4 °C overnight and then analyzed in the Central Laboratory (Department of Clinical Chemistry and Laboratory Medicine, University Hospital Essen). Activities of GLDH, AST and ALT were determined on the ADVIA 1800 Chemistry System (Siemens Healthcare Diagnostics; Eschborn, Germany) by using the respective assay cassettes. Values were normalized to the per-gram weight of the different liver specimens and are presented as means ± standard error. Glucose turnover was calculated by subtracting the initial glucose contents of the culture medium from the concentrations in the perfusates at times of sampling and surplus glucose supplemented during the experiment. Positive values were defined as production or release of a compound, whereas negative values were assumed to reflect consumption.

### Isolation of mtDNA and quantification by real-time qPCR

Mitochondrial DNA (mtDNA) was purified from 1.2 mL perfusate samples taken at 2, 8, 8–19, 19, 24 and 30 h of perfusion using the QiaAmp DNA blood kit (Qiagen; Hilden, Germany) and collected in 60 μL elution buffer according to the supplier’s instructions. Quantitative real time PCR was performed with the Quanti-Fast SYBR Kit (Qiagen) on the C1000 Touch (Bio-Rad; Munich, Germany) – again according to the instructions provided – using the primer pairs F = 5′-ATACCCATGGCCAACCTCCT-3′ and R = 5′-GGGCCTTTGCGTAGTTGTAT-3′ for the *mtND1* gene encoding for NADH dehydrogenase subunit 1, and the primer pairs F = 5′-ATGACCCACCAATCACATGC-3′ and R = 5′-ATCACATGGCTAGGCCGGAG-3′ for the *mtCOX3* gene encoding for cytochrome c oxidase subunit 3^6^. Copy numbers were calculated using a mean amplification efficacy of 88.9% with a threshold of 72.69, and were normalized to the per-gram weight of the liver tissue and 1 mL of perfusate.

### Immunohistochemical staining of cytochrome P450 2E1 and cytochrome c

Sections of embedded liver tissue were deparaffinized. Antigen retrieval was performed by adding citric buffer and heating in a microwave oven for 20 min. Goat-anti-human CYP2E1 antibody (Acris Antibodies GmbH, Herford, Germany) and rabbit-anti-human cytochrome c antibody (Novus Biologicals, Littleton, CO, USA) were diluted 1:250 in phosphate-buffered saline (Gibco) and incubated for 1 h. Immunohistochemistry was performed using the ImmunoCruz goat LSAB staining system (Santa Cruz Biotechnology, Heidelberg, Germany) or STAR 3000a detection kit (AbD Serotec, Düsseldorf, Germany), respectively, according to the supplier’s instructions. Both stains employed 3,3-diaminobenzidine as chromogenic dye. Cell nuclei were counterstained with hematoxylin (Sigma-Aldrich), and digital images were captured on the Axioplan microscope with the camera Axiocam HRc (Carl Zeiss GmbH, Jena, Germany).

### RNA isolation from perfusates and quantification of miR-122 by real-time qPCR

Isolation of miRNA was performed from the outflows obtained at 8–19 h and 19–30 h of perfusion time. For normalization of extracellular miR-122 levels, SV40-miRNA (Qiagen) was added to the samples (20 pmol/1 mL perfusate) prior to the RNA isolation procedure. RNA then was isolated from 300 μl of each perfusate using Qiazol reagent and miRNAeasy Kit (Qiagen) following the instructions of the supplier. Circulating miR-122 levels were quantified as described previously^7^. Briefly, reverse transcription was carried out by means of the miScript Reverse Transcription Kit (Qiagen) followed by qrtPCR using miRNA SYBR Green PCR reagents (Qiagen). Primer sets of miR-122 and SV-40 used for cDNA synthesis and qrtPCR were purchased from Qiagen. Qrt-PCRs were performed as technical triplicates. The levels of miR-122 were determined by absolute quantification using a standard curve and normalized to the spike-in SV40-miRNA.

## Results

### Liver function and general metabolism is diminished by APAP poisoning

**Figure 2:**
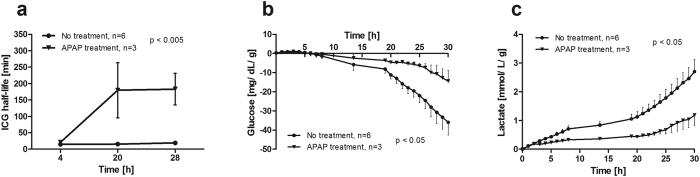
Liver function and metabolic activity during perfusion. (**a**) Half-lives of indocyanine green (ICG) clearance as measure of liver function differed significantly depending on the addition of acetaminophen at 8 h *vs*. untreated control (two-way ANOVA). (**b**) Glucose consumption and (**c**) lactate production were significantly changed towards lower metabolic rate in acetaminophen treated liver specimen compared to untreated liver sections.

Human liver sections were perfused for 30 h in a closed circuit and perfusate was sampled at hourly intervals for blood gas analysis and to determine markers of cell damage and hepatic synthesis capacity. Clearance of indocyanine green (ICG) from the perfusate was measured to assess liver function at 4, 20 and 28 h. Six pieces of human liver tissue without treatment exhibited ICG half-lives of 14.8 ± 0.44, 15.4 ± 1.4 and 19.1 ± 2.9 min, respectively, indicating a nearly stable hepatocellular function during the entire perfusion period (Fig. 2a). Acetaminophen was applied to seven liver specimens (weight 40 ± 3 g) at 6.5 mg/g of liver tissue after 8 h of perfusion and incubated until 30 h. Successful poisoning of the liver was assumed, when ICG half-life at 28 h surpassed triple of the mean half-life of untreated livers (57.3 min). Three tissue samples were successfully damaged (APAP treatment), indicated by turbidity of the perfusate due to cell degradation and ICG half-lives of 21.0 ± 8.0, 179.7 ± 84.2 and 182.9 ± 48.1 min, respectively. In the remaining four specimens acetaminophen caused only weak damage (ICG half-lives: 15.2 ± 2.5, 22.6 ± 4.7; 40.3 ± 6.6 min, respectively; data not shown). Upon APAP poisoning liver sections showed significantly lower glucose consumption (Fig. 2b) and lactate production rates (Fig. 2c) compared to sections without treatment. Glucose consumption was −35.9 ± 6.66 mg/dL/g for non-treated liver sections and −14.4 ± 5.6 mg/dL/g for APAP-treated ones at 30 h of perfusion. Lactate levels increased to 2.7 ± 0.4 mmol/L/g in the untreated group and to 1.2 ± 0.4 mmol/L/g in the APAP-treated group at the end of perfusion, suggesting higher metabolic rates in untreated liver sections. Notably, the lactate production was already lower prior addition of APAP in the APAP-treated group.

### Hepatocyte synthesis capacity is not affected by APAP poisoning

**Figure 3:**
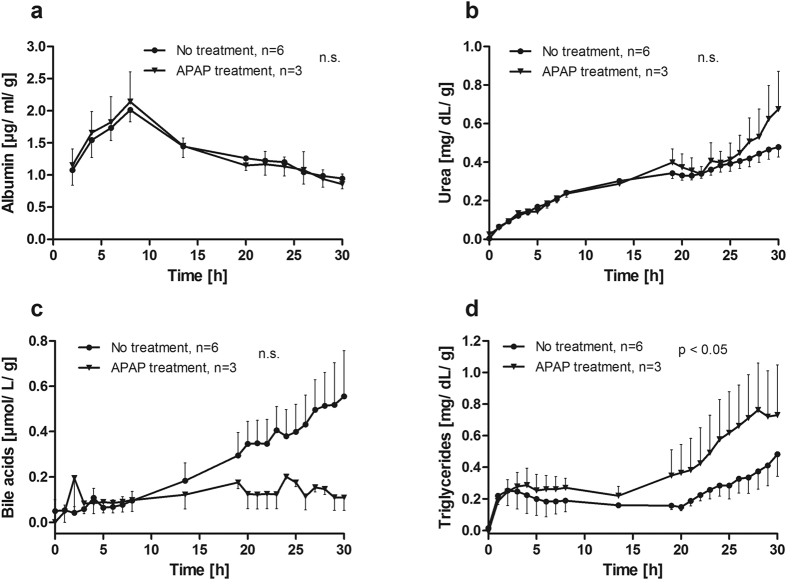
Hepatocyte-specific biosynthesis. Release of (**a**) albumin, (**b**) urea, (**c**) bile acids, and (**d**) triglycerides (TG) into the perfusate were determined. Albumin and urea were continuously released into the perfusate with no difference between APAP-treated and untreated liver specimen. The release of bile acids (**c**) from untreated livers increased over the course of perfusion while it remained at a low level in APAP-treated livers, though this difference did not reach significance. The secretion of triglycerides (**d**) was significantly higher in APAP-treated liver sections, but elevated levels were already detected before APAP application.

The hepatic synthesis capacity was monitored for all perfusion runs performed with or without APAP treatment (Fig. 3). The production rates of albumin (Fig. 3a) and urea (Fig. 3b) were nearly identical during the entire perfusion period for both groups. Higher secretion of bile acids was observed from non-treated liver specimen compared to APAP-treated liver sections starting at 8 h of perfusion. Bile acid concentrations were 5-fold higher in non-treated liver in comparison to APAP treatment at the end of perfusion, though significance was not reached (0.56 ± 0.2 *vs.* 0.11 ± 0.06 μmol/L/g, Fig. 3c). In contrast, significantly higher amounts of triglycerides were released from APAP-treated than from non-treated liver sections during perfusion until 30 h (0.72 ± 0.32 *vs.* 0.48 ± 0.14 mg/dL/g, p = 0.0132, Fig. 3d). However, higher concentrations of triglycerides were observed in the APAP-treated group already prior poisoning.

### Low markers for cell damage in APAP-poisoning suggest a previously impaired hepatocellular condition

**Figure 4:**
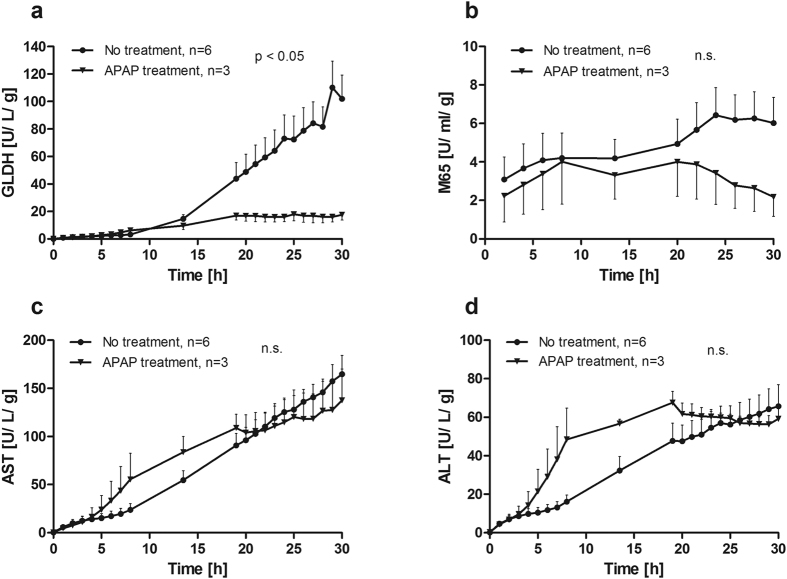
Course of markers for liver cell damage during perfusion. Release of (**a**) glutamate dehydrogenase (GLDH), (**b**) cytokeratin-18 (M65), (**c**) aspartate aminotransferase (AST) and (**d**) alanine aminotransferase (ALT) into the perfusate were detected. More GLDH and M65 were released from untreated liver specimen compared to acetaminophen treated (APAP) livers while AST and ALT did not differ between the two groups (two-way ANOVA).

Release of intracellular compounds into the perfusate was assessed to determine cell damage. For glutamate dehydrogenase (GLDH, Fig. 4a) as a marker of severe cell destruction^8^ an increase to 101.9 ± 17.3 U/L/g was observed in the group without treatment at 30 h, while the group with APAP treatment exhibited significantly lower levels (17.6 ± 3.9 U/L/g). The release of Cytokeratin-18 (M65, Fig. 4b) as marker for overall cell death reached 6.02 ± 1.33 U/mL/g at 30 h. The M65 levels for the APAP-treated group decreased to 2.18 ± 1.00 U/ml/g at the end of perfusion. The releases of aspartate and alanine aminotransferases (AST, Fig. 4c; ALT, Fig. 4d) did not differ between the groups. However, ALT and AST concentrations were already higher prior to addition of APAP in livers with APAP toxicity. These findings suggest that pronounced APAP-induced liver damage occurs in liver specimens with initially impaired hepatic status.

**Figure 5:**
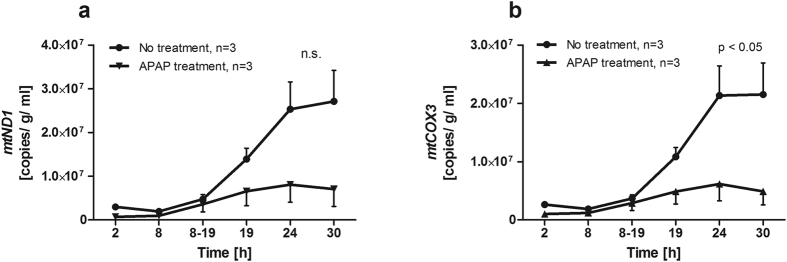
Mitochondrial DNA as marker for liver injury during course of perfusion. Release of DNA of the two mitochondrial genes *MT-ND1* (**a**) and *MT-COX3* (**b**) into the perfusate was quantified. About three times more mtDNA was found in the perfusate of untreated liver specimen compared to acetaminophen (APAP) treated ones at 24 and 30 h of perfusion. The difference was significant for *MT-COX3* DNA (two-way ANOVA).

Apart from GLDH, the release of mitochondrial DNA (mtDNA) into the perfusate was detected as additional marker for severe cell destruction including mitochondrial damage^6^. MtDNA was isolated from the perfusate at 2, 8, 8–19, 19, 24, and 30 h. Free copies of *MT-ND1* (coding for NADH dehydrogenase) and *MT-COX3* (coding for cytochrome c oxidase) were quantified by qrtPCR. Copy numbers for both genes were about 3 times higher after 24 and 30 h of perfusion time in liver sections without treatment compared to those with APAP treatment (Fig. 5a,b).

### Histochemical tissue characterization and CYP2E1 localization indicate mitochondrial damage without impairment of cellular integrity

**Figure 6:**
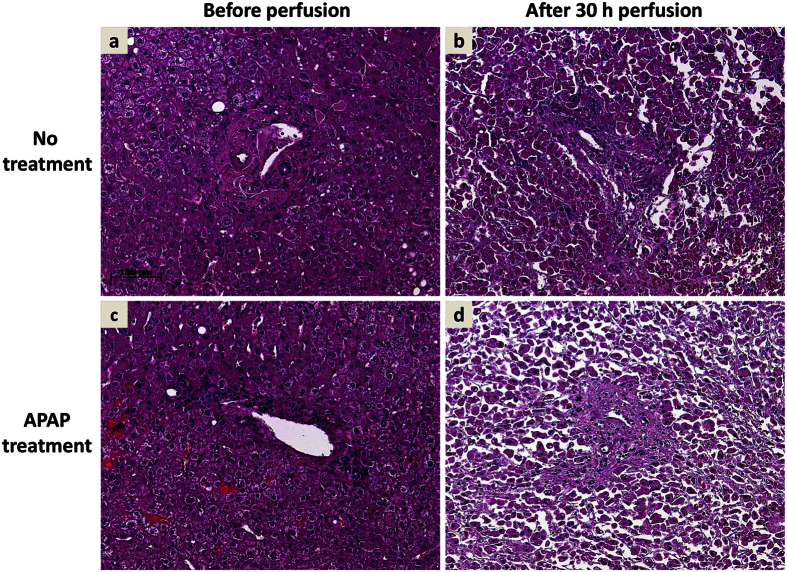
Liver perfusion causes tissue deterioration, which was aggravated by APAP poisoning. Depicted are representative hematoxylin & eosin staining of matched liver tissue prior perfusion (**a,c**) and after 30 h of perfusion without treatment (**b**) or APAP treatment (**d**). While perfusion leads *per se* to deterioration of tissue integrity and cellular damage, large areas of confluent hepatocytes seem morphologically intact (**b**). Perfusion with APAP results in widespread tissue disruption, necrosis, and singularized cells without hepatocellular morphology. Sections a/b and c/d derive from the same liver specimen.

Tissue structure and integrity were visualized by HE staining. Depicted are two representative perfusion experiments with non-treated (Fig. 6a,b) and APAP-treated (Fig. 6c,d) liver specimen before and after perfusion. Tissue sampled prior perfusion (a, c) showed largely intact liver parenchyma without visible cell death. After 30 h of perfusion without treatment (Fig. 6b) the cell structure exhibited lesions with areas of mostly intact contiguous liver cells. In contrast, after 30 h perfusion with APAP treatment (Fig. 6d) the parenchyma was dissolved and mainly singularized hepatocytes with many non-parenchymal cells interspersed were observed.

To assess expression of cytochrome P450 2E1 (CYP2E1), the isoenzyme producing the reactive metabolite NAPQI from APAP, immunohistochemistry was performed. Homogeneously distributed expression was found in nearly all cells prior perfusion (Supplemental Fig. 1a,c), suggesting localization in specific subcellular compartments. While a similarly homogeneous distribution and intensity was found after perfusion, no intracellular structures could be identified in the staining patterns (Supplemental Fig. 1b,d). No differences in the amount or localization of CYP2E1 expression could be identified between non-treated and APAP-treated liver sections. Also no differences between successfully poisoned APAP-treated liver sections and mildly injured APAP-treated specimen were observed (data not shown).

Immunohistochemical staining for cytochrome c as mitochondrial marker also displayed discrete intracellular localization prior perfusion. Consistent expression was observed in most liver cells resulting in a network within the cytoplasm (Supplemental Fig. 2a,c). After perfusion more diffuse patterns were found within the cytoplasm of untreated liver specimen indicating release of cytochrome c by destruction of mitochondria (Supplemental Fig. 2b,d). The overall intensity of cytochrome c staining seemed weaker in APAP-treated livers (Supplemental Fig. 2d), suggesting loss of cytochrome c from the majority of the cells.

### APAP poisoning leads to elevated release of microRNA-122 into the perfusate

**Figure 7:**
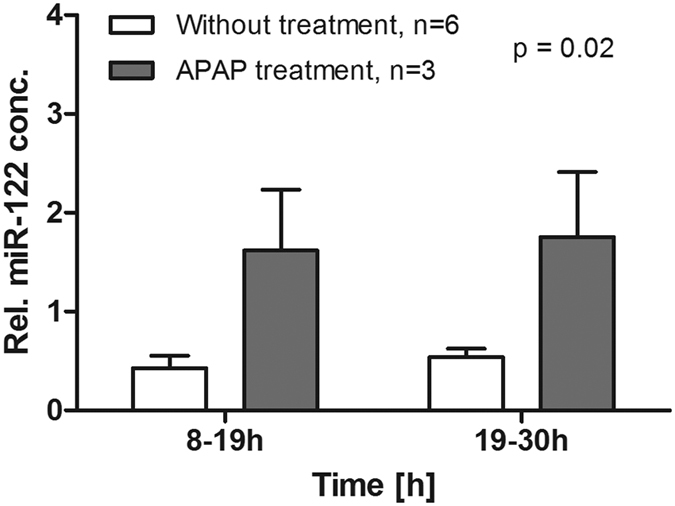
Release of miR-122 into perfusate was elevated in APAP poisoning. Concentrations of miR-122 (normalized to spike in SV-40 miRNA), an early marker of liver damage in acute liver failure, were significantly higher for APAP-treated liver sections than for untreated (2-way-ANOVA) as measured in both outflows collected from 8–19 h and from 19–30 h perfusion time.

Severe liver cell injury in humans is also accompanied by the release of microRNAs into serum^9^, and miR-122 has been associated with drug-related hepatotoxicity^10^. The concentrations of microRNA-122 (miR-122) were measured in the outflows 1 (collected from 8 to 19 h) and 2 (19–30 h) representing the early and late phase after APAP intoxication, respectively (Fig. 7). Relative miR-122 concentrations in non-treated liver specimen were 0.43 ± 0.13 in outflow 1 and 0.54 ± 0.09 in outflow 2 representing constant low levels during the entire perfusion time. The miR-122 levels increased significantly to 1.62 ± 0.62 in outflow 1 and 1.76 ± 0.66 in outflow 2 (p = 0.02) for liver specimen with successful APAP poisoning. In APAP-treated livers with only weak damage miR-122 remained low in outflow 1 (0.47 ± 0.35) and was only elevated in outflow 2 (1.29 ± 0.42).

## Discussion

The presented human *ex vivo* model has been shown to induce reproducible APAP-induced liver damage within 12–20 h. Main outcomes of successful APAP poisoning were impaired hepatocyte metabolism, signs of specifically mitochondrial damage and increased release of miR-122. In contrast, liver synthesis parameters and classic surrogate markers of cell death were hardly affected by APAP poisoning. The presented data also indicate that the perfusion system mimics human variability in reaction to drugs. The applied amounts of APAP (6.5 mg/g corresponding to approx. 6.9 mM for a liver piece of 40 g) were the lowest dose necessary to provoke liver injury. This dose would correspond to 8.1 g of APAP when extrapolated to an adult human being with a liver weight of approx. 1.25 kg. For individuals with additional risk factors or previous liver injury this amount may induce ALF, whereas healthy subjects may not be affected even by larger amounts. In a mathematical model of APAP toxicity increased formation of protein-adducts already occurred at a dose of 4.54 g^11^. As intoxication was effectively achieved in 43% of APAP-treated livers in our system, actual inter-individual differences seem to be well reflected. For future works, donor associated factors will be investigated more thoroughly to elucidate such differences.

ICG clearance was applied as main outcome measure, as it has been shown to correlate with the extent of hepatic necrosis in murine models of APAP toxicity^12^. Furthermore, a recent study demonstrated a better accuracy to predict 3-month mortality in ALF patients for a combination of ICG testing and the model-for-end-stage liver disease score than conventional scores^13^. Thus, we are confident that ICG is sufficient to determine liver damage in the presented system even though transaminases did not indicate differences in damage. This is corroborated by the observation that a relative small area of viable cells can maintain ICG clearance (data not shown), requiring nearly complete depletion of hepatocytes to prolong half-lives above 100min.

Effects of APAP poisoning on general metabolism and hepatic synthesis capacity demonstrate operation of hepatocytes in a normal physiological manner. Similar courses of albumin and urea concentrations in perfusates for non-treated and APAP-treated liver sections indicate similar protein production and degradation rates in both groups. Nevertheless, lower lactate production and higher triglyceride secretion in liver specimen with successful APAP poisoning before addition of APAP suggest an already impaired metabolism in these specimen. This may have contributed to the more severe damage due to APAP, which is actually supported by clinical data^14^,^15^.

Despite clear indications of diminished hepatocyte functionality and dramatically increased tissue disruption in APAP poisoning shown by HE, release of damage markers was unexpectedly low in APAP poisoned livers. Release of classic liver injury markers did not differ between untreated and APAP-poisoned livers. Though, higher release of GLDH and M65 and lower release of mtDNA from untreated liver sections were observed. This seemingly contradicts reports that higher GLDH and mtDNA values in patients’ sera correlated with poor survival after acetaminophen-induced ALF^6^. Though, higher GLDH as well as M65 concentrations in untreated liver sections could be explained by a larger number of intact cells in untreated liver tissue. In contrast, liver sections effectively damaged by APAP might have been susceptible due to already reduced numbers of fully functional hepatocytes. This would be supported by diminished release of damage markers and reduced metabolic activity, as observed in the presented experiments. Findings in patients with ALF support this hypothesis as high levels of transaminases correlated with spontaneous survival^16^. This indicates higher numbers of functional hepatocytes on the one hand release larger amounts of various molecules, including damage markers, and on the other hand allow restoration from acute liver damage. Conversely, reduced numbers of fully intact hepatocytes lead to lower absolute amounts of damage markers but also to a worse outcome. Again, this reflects the clinical reality, since in cases with acute-on-chronic liver failure usually much lower levels of liver transaminases are observed than in ALF^17^. An alternative explanation for this observation could be rapid intracellular degradation of these molecules during severe cellular damage prior release into the perfusate or serum. Various outcomes after APAP treatment have also been reported from different *in vivo* and *in vitro* models^18^, and our findings might be a particular characteristic of the *ex vivo* perfusion system. Immunohistochemical assessments of CYP2E1 and cytochrome c suggest disruption of intracellular structures, in particular of mitochondria, which would be supported by mitochondrial DNA found in perfusate. Early depletion of mitochondria or even reduced numbers of mitochondria prior perfusion might explain the seemingly stronger damage visible in cytochrome c staining of APAP-poisoning contrasted with higher mtDNA in untreated livers. This hypothesis will be addressed in future works.

miR-122 is elevated in human sera during liver injury, especially due to pharmacological damage^19^,^20^. The release of miR-122 in the presented system supported the findings from ICG clearance at 20 and 28 h of perfusion, indicating impaired hepatocellular function. In human ALF release of miRNAs precedes the release of transaminases^21^. Thus miRNAs might be excreted from cells before necrosis leads to membrane disruption and release of other liver injury markers into the perfusate. This could imply that the observed time frame of liver damage in our system may be too short, to detect alterations of classic liver damage markers. Future works will address this question and options for further expansion of perfusion time will be tested.

The usability of this perfusion model is limited by availability of human liver and by the technical complexity, but the advantages may outweigh this in some cases. Animal models cannot always indicate hepatotoxicity of novel drugs due to differences in drug metabolism and mostly homogeneous environmental as well as genetic conditions in inbred animal strains. In the here presented model effects of new active compounds can be assessed in a human based environment, reflecting inter-individual differences including conditions of higher susceptibility. Findings from this system could contribute to drug safety of novel substances. Compared to cell cultures, conserved hepatic functionality and interaction of hepatocytes with non-parenchymal cells are included providing results closer to the human *in vivo* situation. A recently published system employing spheroids of primary human hepatocytes could also impressively mimic liver function, exhibited hepatotoxic sensitivity and enabled co-culture with non-parenchymal cells^22^. Though, despite of a longer cultivation time compared to the here presented system, fewer cells in a non-native architecture were seeded. While the spheroid system could be more readily available for reproducible drug testing, the here presented system could give a more comprehensive view on liver physiology and mechanistic pathways in disease states and toxicity.

## Additional Information

**How to cite this article**: Schreiter, T. *et al*. Human *Ex-Vivo* Liver Model for Acetaminophen-induced Liver Damage. *Sci. Rep.* **6**, 31916; doi: 10.1038/srep31916 (2016).

## Supplementary Material

Supplementary Information

## Figures and Tables

**Table 1:** Clinical data of liver specimen received from partial hepatectomies for *ex-vivo* liver perfusion.

Treatment	Wet Weight [g]*	Gender	Age	Underlying Disease	Surgical Intervention
w/o	40	male	75	Hepatocellular carcinoma	Right-side hemihepatectomy
w/o	52	female	69	Metastasized colon carcinoma	Right-side hemihepatectomy
w/o	31	male	74	Cholangiocellular carcinoma	Right-side hemihepatectomy
w/o	29	male	61	Hepatocellular carcinoma	Trisegmentomy V+VI+VII
w/o	40	male	52	Klatskin tumor	Extended right-side hemihepatectomy
w/o	30	female	78	Metastasized colon carcinoma	Bisegmentomy II+III
APAP	39	male	43	Metastasized caecum carcinoma	Right-side hemihepatectomy
APAP	40	male	57	Metastasized rectal carcinoma	Right-side hemihepatectomy
APAP	39	male	44	Metastasized rectal carcinoma	Right-side hemihepatectomy

^*^Final weight of the employed liver specimen.
