# Supplementary material for: Human Ex-Vivo Liver Model for Acetaminophen-induced Liver Damage
Source: Sci Rep. 2016 Aug 23;6:31916. doi: 10.1038/srep31916 (PMC4994032; doi:10.1038/srep31916)

Human Ex-Vivo Liver Model for Acetaminophen-induced Liver Damage

Thomas Schreiter, Jan-Peter Sowa, Martin Schlattjan, Jürgen Treckmann, Andreas Paul, Karl-Heinz Strucksberg, Hideo A. Baba, Margarete Odenthal, Robert K. Gieseler, Guido Gerken, Gavin E. Arteel, Ali Canbay

Supplemental Figure 1: Immunohistochemical staining for cytochrome P450 2E1. Non-treated (a, b) and APAP-treated (c, d) liver sections were stained before and after 30 h of perfusion. Image-pairs a-b and c-d originate from the same experiment. Prior perfusion localization of cytochrome P450 2E1 within subcellular compartments was observed. After perfusion unspecific staining of the whole cytosol was present, suggesting destruction of intracellular organelles. No difference between untreated or APAP-treated liver specimen was detectable.

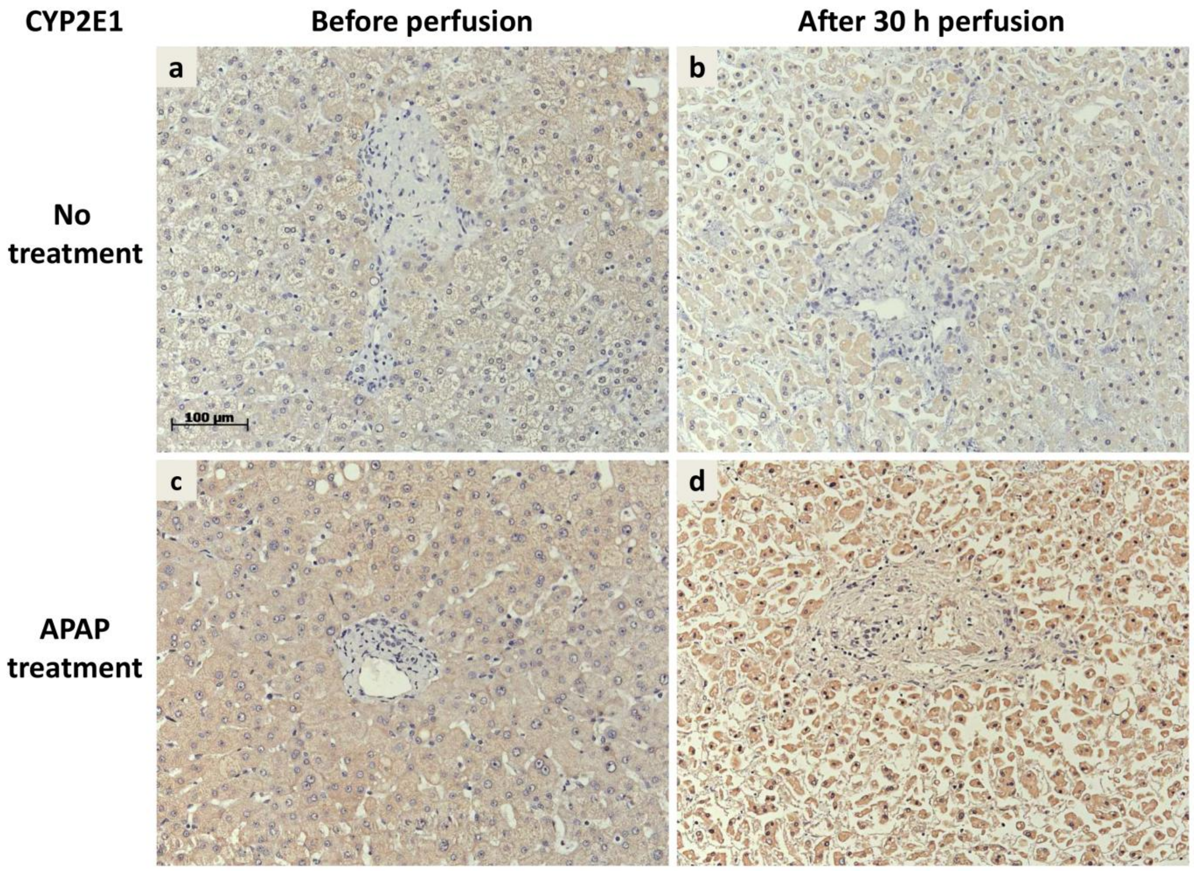

Supplemental Figure 2: Immunohistochemical staining for cytochrome c. Non-treated (a, b) and APAP-treated (c, d) liver sections were stained before and after 30 h of perfusion. Image-pairs a-b and c-d originate from the same experiment. Prior perfusion localization of cytochrome c within subcellular compartments, probably mitochondria, was observed. After perfusion unspecific staining of the whole cytosol was present and staining intensity was reduced, suggesting destruction of mitochondria and possible release of components into the perfusate. While staining intensity was weaker in APAP-treated liver tissue, no statistical difference between untreated or APAP-treated liver specimen could be determined by digital quantification (not shown).

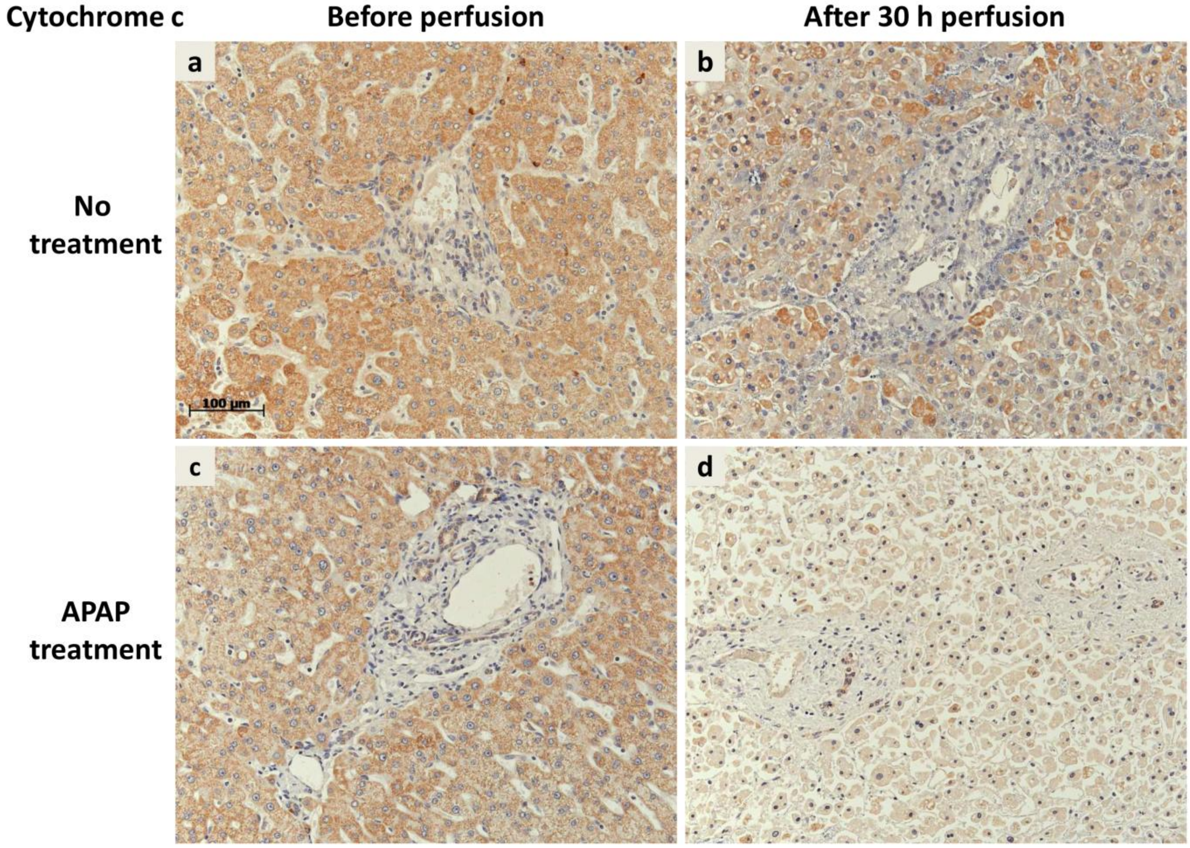

Supplement: Supplementary Information [file srep31916-s1.pdf]
